# Supplementary material for: Enhanced Proteomics Analysis with a Novel Recombinant Chymotrypsin Analogue Engineered for High Cleavage Specificity
Source: J Proteome Res. 2026 Apr 10;25(5):2511–9. doi: 10.1021/acs.jproteome.5c01262 (PMC13140120; doi:10.1021/acs.jproteome.5c01262)
Supplement: Supplementary file 1 [file pr5c01262_si_001.pdf]

Supplementary Information: Enhanced Proteomics Analysis with a Novel Recombinant Chymotrypsin Analogue Engineered for High Cleavage Specificity

Kish R. Adoni[1†]; Jonathan E. Ditcham[1]; Alba Katiria González Rivera[4]; Georgina H. Charlton[1]; Sergei Saveliev[4]; Konstantinos Thalassinos[1,2,3*]; Riccardo Zenezini Chiozzi[1,3*]

1. Institute of Structural and Molecular Biology, Division of Biosciences, University College London, London WC1E 6BT, United Kingdom

2. Institute of Structural and Molecular Biology, School of Natural Sciences, Birkbeck College, University of London, London WC1E 7HX, United Kingdom

3. University College London Mass Spectrometry Science Technology Platform, Division of Biosciences, University College London, London, UK

4. Promega Corporation, Madison, WI, WI 53711, United States

KEYWORDS Chymotrypsin, Proteomics, Protein Digestion, Sample Preparation, Missed Cleavages

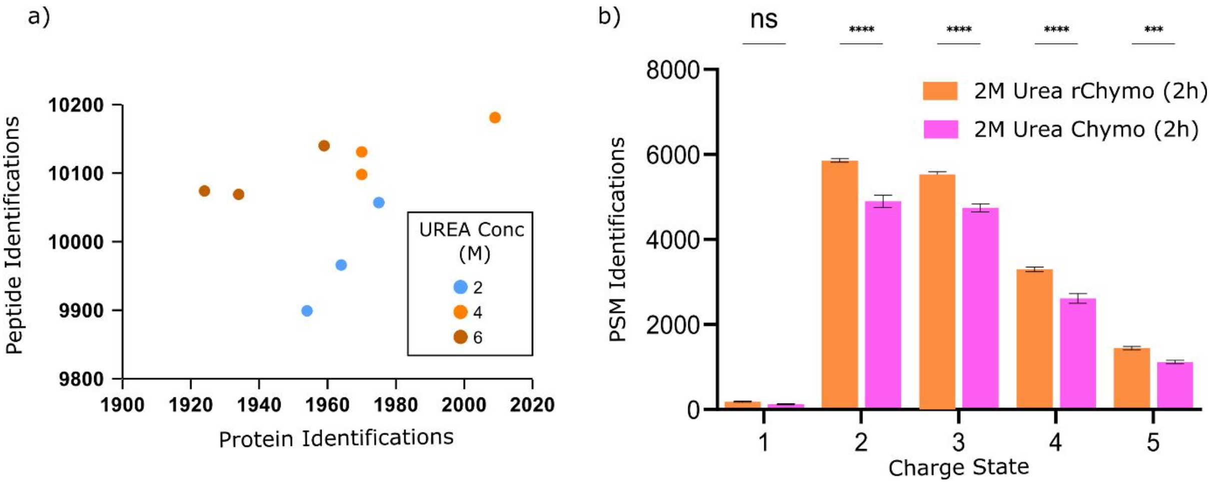

Fig. S1: (a) Peptide and protein identifications for 2M, 4M and 6M urea concentration (2h incubation) for rChymoSelect. (b) Number of PSMs for rChymoSelect vs standard chymotrypsin for 2^+ - 5^+ charge-state

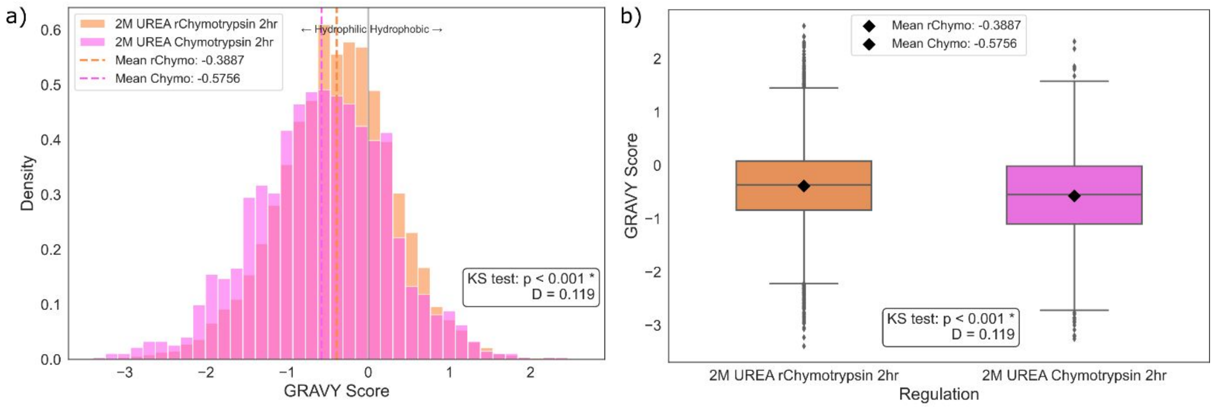

Fig. S2 Comparison of GRAVY scores of uniquely identified peptides with rChymo (orange) and standard chymotrypsin (orange), visualised as a histogram (a) and box plot (b).

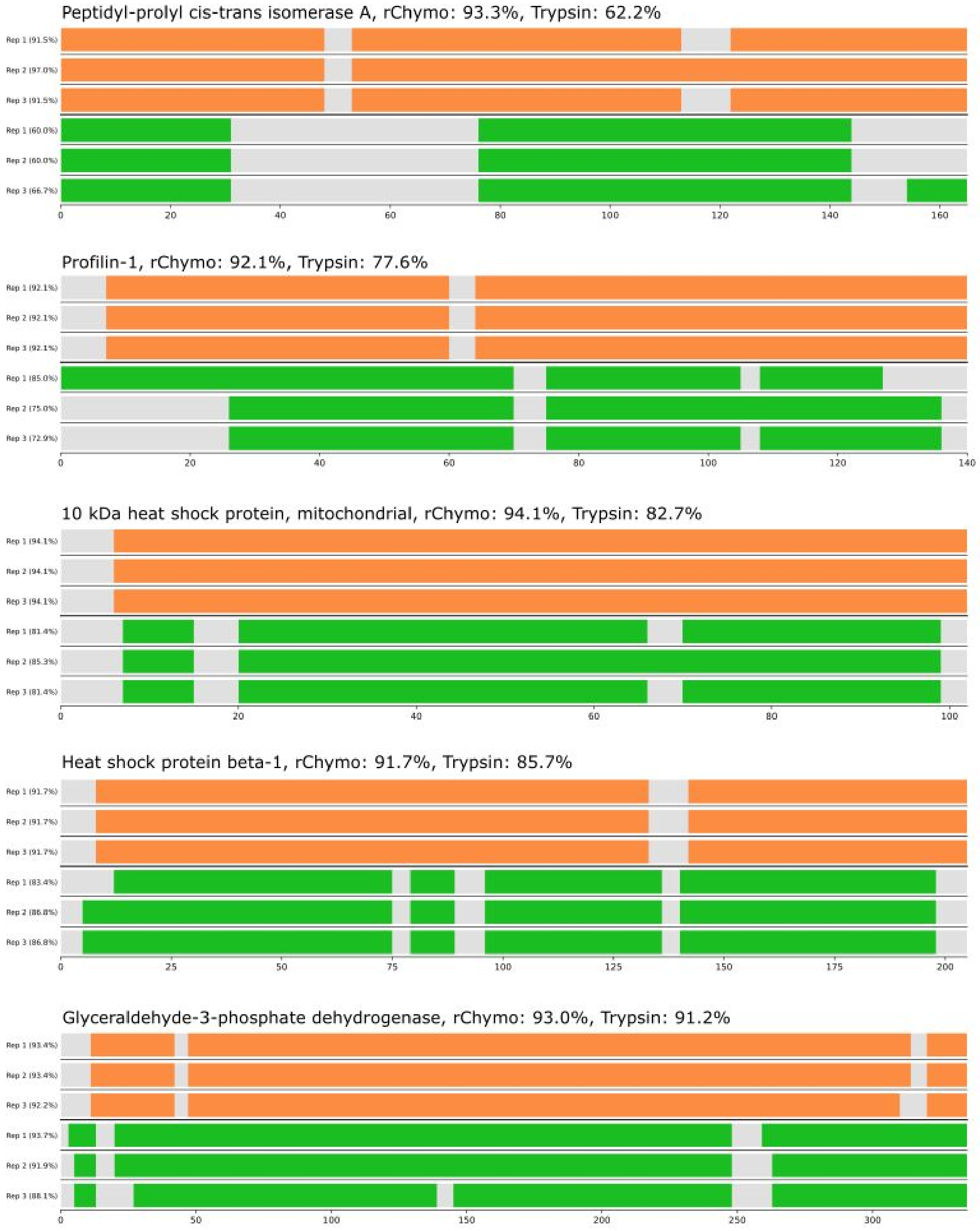

Fig. S3: Sequence coverage for rChymoSelect (orange) and Trypsin (green) for selected proteins with sequence coverage 90 – 100% with rChymoSelect.
